# Medicolegal Considerations with Intravenous Tissue Plasminogen Activator in Stroke: A Systematic Review

**DOI:** 10.1155/2013/562564

**Published:** 2013-09-04

**Authors:** Archit Bhatt, Adnan Safdar, Dhara Chaudhari, Diane Clark, Amber Pollak, Arshad Majid, Mounzer Kassab

**Affiliations:** Providence Brain and Spine Institute, Portland, OR 97225, USA

## Abstract

*Background.* Intravenous tPA (tissue plasminogen activator) therapy remains underutilized in patients with Acute Ischemic Stroke (AIS). Anecdotal data indicates that physicians are increasingly liable for administering and for failure to administer tPA. *Methods.* An extensive search of Medline, Embase, Westlaw, LexisNexis Legal, and Google Scholar databases was performed. Case studies that involved malpractice litigation in ischemic stroke and thrombolytic therapy were analyzed systematically. *Results.* We identified 789 ischemic stroke litigation cases, of which 46 cases were related to intravenous tPA and stroke litigation. Case descriptions of 40 cases were available. Data for verdicts were available for 38 patients. The most frequent plaintiff claim was related to failure to administer intravenous tPA (38, 95%). Only 2 (5.0%) claim involved complications of treatment with tPA. Hospitals were defendants in majority of the 36 cases. Physicians were involved in 33 cases. While ED physicians were involved in 25 (60.52%) cases, neurologists were involved in 8 (20.0%) cases. There were 26 (65%) defendant-favored and 12 (30%) plaintiff-favored verdicts. *Conclusion.* Physicians and hospitals are at an increased risk of litigation in patients with AIS when in IV-tPA is being considered for treatment. While majority of the cases litigated were cases where tPA was not administered, only about 1 in 20 cases was litigated when complications occurred.

## 1. Introduction

Acute Ischemic Strokes (AIS) is the number one cause of morbidity and third leading cause of mortality in the developed world behind heart disease and cancer. Approximately 795,000 cases of strokes occur annually in the United States, of which 610,000 are first ever strokes. Prior to 1995, there was no FDA-approved thrombolytic treatment available for AIS. Between 1991 and 1995, a NINDS sponsored randomized trial [1] was conducted to assess the safety and efficacy of recombinant tissue plasminogen activator (tPA) in patients with AIS, within 3 hours of stroke onset. The results showed that tPA-treated stroke patients were 32 percent more likely to show minimum or no disability at 3 months (odds ratio 1.7, CI 1.2–2.6, NNT 8, NNH 16, and ARR 12%), compared to patients who did not get tPA. Symptomatic intracerebral hemorrhage within 36 hours after the onset of stroke occurred not only in 6.4 percent of patients given tPA but also 0.6 percent of patients given placebo (*P* < 0.001). Mortality at three months was 17 percent in the tPA group and 21 percent in the placebo group (*P* = 0.30). Over subsequent years, two trials [2, 3] evaluated tPA within 0–6 hours, showing that tPA isnot efficacious in the expanded time window. However, tPA has recently been shown to be effective in a selected group of stroke patients between 0 and 4.5 hours [4].

A joint report by AHA stroke council and AAN quality standards committee states that tPA should be considered in patients with ischemic stroke within 3 hours [5]. In 2002, the American Academy of Emergency Medicine [6] position statement raised concerns about the risk and benefit ratio of tPA in stroke and debate over whether tPA should be considered standard of care. The statement argued that the National Institute of Neurological Disorders and Stroke (NINDS) study suggested that 8 out of 18 (44%) stroke patients who receive tPA according to a strict protocol recover by three months after the event without significant disability. Whereas, 6 out of 18 (33%) stroke patients (one-third) recover substantially, regardless of treatment, they also indicated that 1 out of 18 patients have a symptomatic bleeding complication.

Malpractice is defined as “the failure to meet a standard of care or standard of conduct that is recognized by a profession reaches the level of malpractice when a client or patient is injured or damaged because of error.” The burden of the proof or preponderance of evidence is on the plaintiff, in any medical malpractice litigation. In other words, if a jury believes there is at least 51 percent likelihood that a defendant was negligent or liable, the plaintiff has met its burden of proof and will prevail. This is particularly helpful when juries cannot decide between the testimonies of two expert witnesses presenting opposite opinions or views.

In the United States, AIS is the number one cause of disability and morbidity thus attracting medical litigation. A study reviewing malpractice cases in New York State showed that severity of the patient's disability, not the occurrence of an adverse event due to medical negligence, was predictive of payment to the plaintiff [7]. Frivolous medical malpractice lawsuits are common; consequently, exorbitant amount of human and financial resources is utilized, even if the case eventually is ruled in favor of the defendant [8]. 

According to the 2009 PIAA Risk Management Review: Neurology Edition (available at http://www.piaa.us) [9], for the year 2008, neurology and neurosurgery had the highest average indemnity of the 28 specialties included in the PIAA review. In the case of tPA and stroke, medical litigation works as a double-edged sword. Frequently reasons cited for litigation in the court of law include lost opportunity to give tPA or adverse events related to tPA. Recent reviews [10, 11] have emphasized that physicians are at risk for malpractice suits both when administering and not administering tPA to AIS patients. Disinclination to use tPA by physicians for legal or clinical reasons may potentially lead to medical malpractice litigation. 

To date, no systematic reviews have been performed, which evaluate malpractice and thrombolytic therapy in ischemic stroke patients. The objective of this review is to do a systematic evaluation of malpractice cases published in major medical and legal databases.

## 2. Methods

### 2.1. Protocol

**Figure 1:**
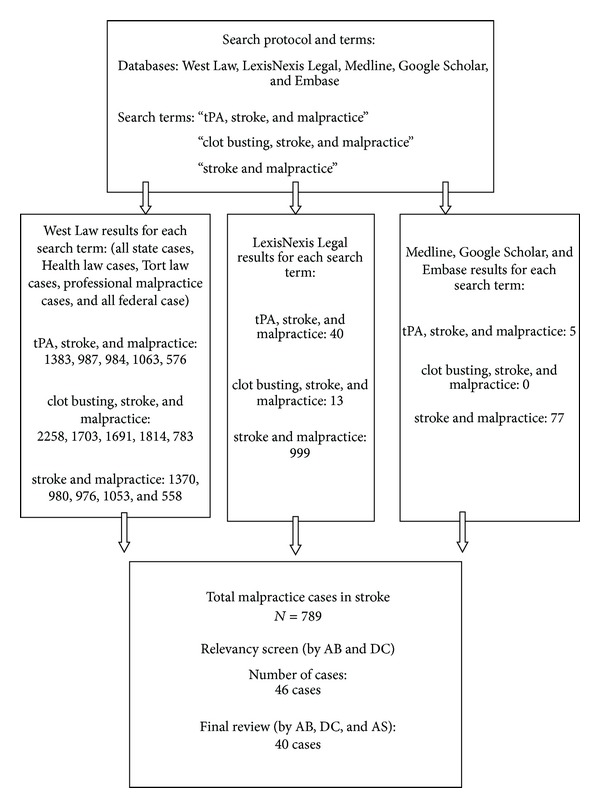


A robust systematic review protocol (Figure 1) was prepared. We searched Medline, Embase, Google Scholar, LexisNexis Legal, and Westlaw databases in order to identify cases, which involved malpractice litigation and tPA in ischemic stroke. LexisNexis Legal is a comprehensive database, which covers state as well as federal law cases in the USA Westlaw is a legal attorney database providing information on cases noted by practicing attorneys. In an attempt of completeness, we searched all state case, Health law case, Tort law case, Professional Malpractice case, and all federal case databases that fall within the Westlaw database.

### 2.2. Search Terms

We used 3 different search terms to be specific to our search, namely, “tPA, stroke, and malpractice,” “clot busting, stroke, and malpractice,” and “stroke and malpractice.” 

### 2.3. Search Results in Each Database

Using the terms “tPA, stroke, and malpractice” yielded 5 results in Medline, 40 in LexisNexis Legal, and 1383, 987, 984, 1063, and 576 results in All State law, Health law, Tort Law case, Professional Malpractice case, and all federal case databases, respectively (all of which are part of Westlaw database). The term “clot busting, stroke, and malpractice” did not show up any results in Medline but gave 13 results in LexisNexis Legal, and 2258, 1703, 1691, 1814, and 783 in All State law, Health Law, Tort law, Professional malpractice case, and all federal case databases of Westlaw. The term “stroke and malpractice” gave us 77 results in Medline, 999 in LexisNexis Legal, and 1370, 980, 976, 1053, and 558 search results in All State Law, Health Law, Tort Law, Professional Malpractice case, and all federal case databases. The Google scholar and Embase database results were subsumed in the Medline search results.

Finally, we identified 789 stroke cases with malpractice suits. AB and DC reviewed case summaries of all cases with the objective to retrieve malpractice lawsuits involving use or nonuse of intravenous tPA in AIS. Finally, 46 cases were identified, and full review was carried out by three reviewers (AB, DC, and AS).

## 3. Results

We identified 789 ischemic stroke cases involved in litigation. We were able to identify 46 cases related to tPA use in stroke patients between the years 1999 and 2010, of which data was available for 40 cases related to tPA in stroke. However, verdicts were only available for 38 cases. We categorized medico- legal characteristics depending on the malpractice claims and related verdicts (Table 1), hospital/physician characteristics (Table 2), factors favoring defendants (Table 3), and factors favoring plaintiffs (Table 4).

### 3.1. Malpractice Claims

The most frequent malpractice claims by the plaintiff were failure to treat with tPA (28, 70%) and failure to diagnose stroke, claiming a lost opportunity to give tPA (10, 25%). Only 2 (5.0%) patients had claims involved complications of treatment with tPA. Of all cases, where verdicts were available, in 90% cases (*n* = 36) hospital was the defendant. There were 12 (30%) cases where only hospitals were defendants. Out of the 36 (90%) cases involving the hospitals, 28 (75%) were community hospitals, and 9(25%) were university or university affiliated. ED physicians were involved in 25 (60.52%) cases and neurologists participated in 8 (20.0%) cases. In a minority of cases 7 (17.5%), other physicians such as internists, neurosurgeons, and ICU physicians were involved. However, multiple physicians were involved in 11 (27.5% of cases). There were 5 (12.5%) cases where stroke occurred during the hospital stay.

### 3.2. Factors Favoring the Defendant

Factors favoring the defendant incuded proper documentation of contraindications and discussion regarding risks and benefits (50%), expert witness testimony (25%), duration of symptoms beyond 3 hr at the time of presentation (15.6%), informed consent (6.3%), no specific time of onset of symptoms (6.3%), tPA protocol in hospital (9.4%), and tPA not available in hospital (3.1%).

### 3.3. Factors Favoring the Plaintiff

Among all malpractice claims, factors favoring the plaintiff were failure to treat with tPA (67.5%), failure to diagnose (20%), failure to transfer to an institution where thrombolysis can be given (20%), no informed consent or proper documentation regarding contraindication (7.5%), delay in evaluating the patient by a doctor (12.5%) obtaining tests (10%), failure to perform proper medical exam (5%), failure to recommend tPA as a treatment (10%), and complications as a result of tPA treatment (5%). 

## 4. Discussion

Our review indicates that hospitals, ED physicians, neurologists, and sometimes other physicians at the forefront of stroke management are always at risk for medical litigation. It appears from the case descriptions that not treating with tPA (38, 95% cases) is the main causative factor for litigations as 38 out of 40 cases reviewed did not receive tPA (i.e., the standard of care). Reasons of litigation were mostly preventable. Major plaintiff arguments in these cases were failure to diagnose ischemic stroke, inadequate documentation why tPA was not given, failure to transfer to a facility where tPA can be given, and delay in physician or CT evaluation. Our analysis showed that only two cases were related to complications of tPA, out of which 1 was ruled in favor of plaintiff and 1 in favor of defendant. Early data [12] have suggested that only 50% of neurologists were actually giving tPA. Early data also show that tPA is used by less than 2% of the community hospitals [13]; this number has probably increased as a result of comprehensive efforts by JCAHO [10]. Despite ongoing efforts, only 5–10% patients with stroke receive tPA [14]. As a result of perceived complications (e.g., intracranial hemorrhage), surveys have also shown that lack of benefits [15] and medico legal concerns were reported as barrier [11] to give tPA. A review suggested that physician reimbursement for the evaluation and treatment of acute stroke, when compared with other diagnoses commonly treated by neurologists, is relatively low in both the USA and Canada. Health policy decision makers in the USA and Canada should be made aware of the importance of providing a more balanced plan to provide medical care to stroke patients [16]. In addition, the familiarity and comfort among nonneurology physicians (NNPs) with the administration of tPA is still relatively low in rural settings [17]. Our review showed that even hospitalists, neurosurgeons, or PCPs can be defendants, if they are the only physicians taking care of the patient. Out of 6 cases where nonneurology non-ED physicians were involved, 5 were defendant favored compared to 1 plaintiff favored verdict. On a positive note, more recently, telemedicine efforts has been safely instituted in evaluation of AIS and has increased tPA rates in outlying hospitals in an effective and safe manner [18]. In our review, all 3 cases consisting of timely transfer to outside facilities were ruled in favor of the defendants. The most common reason for transfer was absence of neurology expertise and/or tPA protocol in the hospital. Conversely, 8 cases involved a delay in transfer with 3 cases (42%) being ruled in favor of the plaintiff. These methods potentially reduce legal liability especially in transfer cases. Among all cases reviewed, it was noticed that two thirds of the defendants received a favorable verdict. Informed consent, proper documentation of discussion with family, tPA protocol in place, time of transfer, time of onset of symptoms, and timely transfer were important factors in verdicts favoring the defendants. Risks and benefits associated with tPA as well as limited treatment window timeframe should be explained thoroughly, and patients' decision should be properly documented including transfer time. In 90% of cases, a hospital was the defendant. Hospitals, even those of a smaller size, should take initiatives and invest in human resources to facilitate thrombolytic therapy for AIS in the hospital. We found that ED physicians were more likely to be defendants than neurology physicians. However, rate of defendant-favored verdicts was similar on both sides (ED—84% and neurologist—75%). In 1 out of 4 cases, more than one physician is involved. 

Expert witness testimony on the plaintiff side frequently argued that according to NINDS trial, there is a >51 percent chance that the patient will improve if the patient received tPA. However, expert testimony from the defendant side frequently argued that there is only a 32 percent greater chance that the patient would improve. Taking the legal definition of malpractice, the plaintiff needs to prove that the “patient is more likely to improve than not,” and in other words the emphasis is on the chance of improvement and not the actual percentage. In short, in cases where thrombolysis was not given, argument for lack of efficacy of tPA by any defendant was not a very solid argument by defense expert witnesses, and usually these cases were ruled in favor of plaintiffs.

That brings us to the next point, is tPA in fact standard of care? Malpractice claims against physicians are difficult to measure, and physicians might be able to avoid liability by understanding the standard of care regarding tPA. A review has suggested that once an “experimental and risky to give drug” can eventually become a “standard of care” [11]. Emerging local stroke centers in the community have changed the concept of standard of care from nationwide to community and put expectations of elevated criteria on medical personnel and other hospitals in the community [10]. Overall, physicians are judged by standard of practice in their *community* or *similar communities*. However, that concept is changing, and standard of care may be judged by care that could be provided by a physician under *similar circumstances*. 

## 5. Conclusion

Our review also indicates that hospitals and ED physicians are involved in a majority of these litigation cases. The review shows that having hospital-ED-based protocols for tPA administration, transfer protocols to appropriate care facility, clear discussion and documentation of treatment options by physicians, and early involvement of a neurologist may provide optimal milieu for selecting intravenous thrombolysis candidates for patients with AIS. While the majority of the cases litigated were cases where tPA was not administered, only minority of cases were litigated when complications occurred. 

## Figures and Tables

**Table 1:** Malpractice claims.

Claim	Cases (*n*, %)	Verdict in favor of (*n*, %)
Failure to treat with tPA	28, 70%	Of 28 Defendant: 19, 67.9% Plaintiff: 7, 25% NA: 2, 7.1%
Complication as a result of giving tPA	2, 5%	Of 2 Defendant: 1, 50% Plaintiff: 1, 50%
Failure to diagnose	10, 25%	Of 10 Defendant: 6, 60% Plaintiff: 4, 40%

Total claims	40	Defendant: 26, 65% Plaintiff: 12, 30% NA: 2, 5%

**Table 2:** Physician/facility involved.

Faculty/facility involved	Cases (*n*, %)	Verdict in favor of (*n*, %)
ED physician involved	25, 62.5%	Defendant: 21, 84% Plaintiff: 4, 16%
Neurologist involved	8, 20%	Defendant: 6, 75% Plaintiff: 2, 25%
Other (PCP, hospitalist, ICU, and neurosurgeon)	7, 17.5%	Defendant: 5, 71.4% Plaintiff: 1, 14.3% NA: 1, 14.3%
Multiple physicians involved	11, 27.5%	Defendant: 10, 90.9% Plaintiff: 1, 9.1%
Only hospital involved	12, 30%	Defendant: 4, 33.3% Plaintiff: 7, 58.3% NA: 1, 8.3%
Hospital involved	36, 90%	Defendant: 22, 61.1% Plaintiff: 13, 36.1% NA: 1, 2.8%
Type of hospital involved	Of total 36, Community hospital: 27, 75% University Hospital: 9, 25%	Community hospital: defendant: 17, 62.9% plaintiff: 10, 37.03% University hospital: defendant: 5, 55.6% plaintiff: 3, 33.3% NA: 1, 11.1%
In hospital strokes	5 of total 40, 12.5%	Defendant: 2, 40% Plaintiff: 2, 40% NA: 1, 20%

**Table 3:** Frequency of factors favoring defendant.

Factors favoring defendants	Number of cases (total: 32), percentage (%) of cases	Verdict
Documented contraindication/discussion with the family	16, 50%	Defendant: 12, 75% Plaintiff: 4, 25%
Expert witness	8, 25%	Defendant: 8, 100% Plaintiff: 0
Beyond 3 hrs when diagnosed	5, 15.6%	Defendant: 2, 40% Plaintiff: 2, 40% NA: 1, 20%
“TPA protocol in hospital”	3, 9.4%	Defendant: 1, 33.3% Plaintiff: 2, 66.7%
Informed consent	2, 6.3%	Defendant: 2, 100% Plaintiff: 0
Discussion with patient and family	2, 6.3%	Defendant: 2, 100% Plaintiff: 0
No specific time of onset of symptoms	2, 6.3%	Defendant: 2, 100% Plaintiff: 0
tPA not available in hospital	1, 3.1%	Defendant: 1, 100% Plaintiff: 0
Timely transfer to other hospital	3, 9.3%	Defendant: 3, 100% Plaintiff: 0

**Table 4:** Frequency of factors favoring Plaintiff.

Factors favoring plaintiff	Number of cases (total: 40), percentage (%) of cases	Verdict
Failure to treat with tPA	27, 67.5%	Defendant: 18, 66.7% Plaintiff: 7, 25.9% NA: 2, 7.4%
Failure to diagnose	8, 20%	Defendant: 6, 75% Plaintiff: 2, 25%
Failure to transfer	8, 20%	Defendant: 5, 62.5% Plaintiff: 3, 42.9%
Delay in attending patient by ED physician or neurologist	5, 12.5%	Defendant: 3, 60% Plaintiff: 2, 40%
Failure to recommend tPA as a treatment option	4, 10%	Defendant: 4, 100% Plaintiff: 0
Delay in getting test (CT-scan)	4, 10%	Defendant: 3, 75% Plaintiff: 1, 25%
NO informed consent	3, 7.5%	Defendant: 2, 66.7% Plaintiff: 1, 33.3%
Complications of treatment with tPA	2, 5%	Defendant: 1, 50% Plaintiff: 1, 50%
Failure to perform proper complete exam	2, 5%	Defendant: 0 Plaintiff: 2, 100%
